# Early Childhood Fat Tissue Changes—Adipocyte Morphometry, Collagen Deposition, and Expression of CD163^+^ Cells in Subcutaneous and Visceral Adipose Tissue of Male Children

**DOI:** 10.3390/ijerph18073627

**Published:** 2021-03-31

**Authors:** Robert Mujkić, Darija Šnajder Mujkić, Ivana Ilić, Edi Rođak, Antun Šumanovac, Anđela Grgić, Dalibor Divković, Kristina Selthofer-Relatić

**Affiliations:** 1 Department of Anatomy, Histology, Embryology, Pathological Anatomy and Pathological Histology, Faculty of Dental Medicine and Health, Josip Juraj Strossmayer University of Osijek, Crkvena 21, 31000 Osijek, Croatia; rmujkic@fdmz.hr (R.M.); ilovric.os@gmail.com (I.I.); grgic.angel@gmail.com (A.G.); 2 Department of Anatomy and Neuroscience, Faculty of Medicine, Josip Juraj Strossmayer University of Osijek, J. Huttlera 4, 31000 Osijek, Croatia; antun.sumanovac@gmail.com; 3 Clinical Institute of Nuclear Medicine and Radiation Protection, University Hospital Osijek, J. Huttlera 4, 31000 Osijek, Croatia; 4 Department of Histology and Embryology, Faculty of Medicine, Josip Juraj Strossmayer University of Osijek, J. Huttlera 4, 31000 Osijek, Croatia; erodak@mefos.hr; 5 Department for Paediatric Surgery, University Hospital Osijek, J. Huttlera 4, 31000 Osijek, Croatia; divkovic.dalibor@kbco.hr; 6 Department for Cardiovascular Disease, University Hospital Osijek, J. Huttlera 4, 31000 Osijek, Croatia; selthofer.relatic@gmail.com; 7 Department for Internal Medicine, Faculty of Medicine, Josip Juraj Strossmayer University of Osijek, J. Huttlera 4, 31000 Osijek, Croatia

**Keywords:** obesity, early years, collagen, inflammation, macrophages, adipose tissue

## Abstract

Childhood obesity is a complex health problem, and not many studies have been done on adipose tissue remodeling in early childhood. The aim of this study was to examine extracellular matrix remodeling in the adipose tissue of healthy male children depending on their weight status. Subcutaneous and visceral adipose tissue was obtained from 45 otherwise healthy male children who underwent elective surgery for hernia repairs or orchidopexy. The children were divided into overweight/obese (*n* = 17) or normal weight groups (*n* = 28) depending on their body mass index (BMI) z-score. Serum was obtained for glucose, testosterone, triglyceride, total cholesterol, high-density lipoprotein (HDL), and low-density lipoprotein (LDL) measurements. Sections of adipose tissue were stained with hematoxylin and eosin to determine the adipocytes’ surface area, and Masson’s trichrome stain was used to detect the adipocytes’ collagen content. Immunohistochemistry for CD163^+^ cells was also performed. The results showed that male children in the overweight group had higher serum triglyceride levels, greater adipocyte surface area and collagen content in their subcutaneous adipose tissue, more crown-like structures in fat tissues, and more CD163^+^ cells in their visceral adipose tissue than males in the normal weight group. In conclusion, in male children, obesity can lead to the hypertrophy of adipocytes, increased collagen deposition in subcutaneous adipose tissues, and changes in the polarization and accumulation of macrophages.

## 1. Introduction

The understanding of body fat distribution, structural complexity, function, and physiology represents the very first step in the comprehension of the varying degrees of connection between body mass index (BMI), adiposity, morbidity, and mortality [1]. A decreased or increased caloric intake can drastically affect adipose tissue (AT) and its structural integrity, and trigger multiple events that are mutually connected and known as adipose tissue remodeling [2]. AT remodeling is characterized by processes of inflammatory cell infiltration, the production of proinflammatory cytokines (IL-1, IL-6, TNF-α, TGF-β, IL-18), an enhanced angiogenesis, and an increased deposition of extracellular matrix (ECM) proteins that actively lead to morphological changes in AT [3]. The term “adipose tissue remodeling” is used by some scientists to describe changes that can occur in the cellular composition of stromal cell tissue in response to AT expansion or contraction due to alterations in nutritional intake, while others use the phrase “remodeling” to describe the decomposition and deposition of noncellular components that contribute to the architecture of AT [4,5]. 

The ECM is a key component of AT architecture and is necessary to maintain the balance and integrity of the tissue structure itself. The formation, maintenance, and degradation of the ECM in AT is regulated by many adipose tissue components, including adipocytes and even inflammatory cells [2]. The mechanisms involved in AT expansion in obesity are not fully balanced, and patterns of fat storage and expansion vary greatly from person to person. That difference leads to variations in the onset of metabolic diseases in individuals with the same degree of obesity [6,7]. In obese people, there is an increased deposition of connective tissue (collagen) in the ECM and disturbances in the morphology and architecture of AT. The predominant accumulation of collagen fibers, which is a hallmark of fibrosis development, is accompanied by smoldering inflammation characterized by the infiltration of inflammatory cells with the simultaneous production of proinflammatory cytokines that together lead to AT fibrosis [8].

Collagens are the largest group of ECM proteins and their structure and integrity is strictly controlled at the molecular level [9]. They are the main noncellular component of the ECM, and they contribute significantly to the total amount of noncellular adipose tissue mass. Collagen in the ECM of adipose tissue is primarily produced by adipocytes but can also be produced by preadipocytes, fibroblasts, and endothelial cells, and it contributes to cell adhesion, migration, differentiation, morphogenesis, wound healing, and scar formation in AT. Among several types of collagen, collagen IV is the main component of the basement membrane of every adipocyte and is very important for adipocytes’ survival [8,10,11].

The association between fibrosis and AT inflammation is very complex and intertwined, but, although inflammation can lead to fibrosis, fibrosis can occur regardless of inflammation [12]. Although adipocytes are irreplaceable in their role of controlling local changes in their environment, macrophages also play an important role in the AT remodeling process [13]. In studies with obese mice, an increased accumulation of macrophages in adipose tissue was observed, which is a similar mechanism as that which occurs in obese people. In studies with rodent models, obesity is most commonly caused by a high-fat diet (HFD) and leads to a change in macrophages’ phenotypes in AT, from M2-polarized macrophage types in lean animals to M1-polarized macrophage types in obese animals [14,15]. Chronic inflammation occurs in the AT of obese individuals and is histologically presented by crown-like structures (CLS), which consist of macrophages surrounding dead adipocytes [16,17].

Very little is known about the ECM and AT remodeling processes due to obesity in young children, and obesity in children is increasing. To date, there have been only a few studies about AT remodeling in early childhood using AT samples taken during abdominal surgery [18,19], and the reasons could be ethical or approval problems or other difficulties that can occur in the process of tissue sample acquisition from children.

The aim of this preliminary research was to examine if there were differences between the collagen deposition and the number of CD163^+^ cells (representing M2 macrophages) in the subcutaneous AT (SAT) and visceral AT (VAT) of healthy male children depending on their changes in weight status. Because of the difficulties in obtaining tissue samples from children, we hoped that this research would be a good starting point for further studies with larger sample sizes, so even more detailed explorations could be done to determine a hypothetical link between some early symptoms of metabolic syndromes in childhood and enable the prevention of more complicated metabolic diseases that could occur in older age.

## 2. Materials and Methods

### 2.1. Patients and Tissue Collection

This study was based on 45 otherwise healthy male children aged from 2 to 16 years that were hospitalized for elective abdominal surgery at the Department of Pediatric Surgery of the University Hospital Osijek from June 2018 to December 2019. Tissue samples from subcutaneous and visceral fat depots were collected during surgery from children having hernia repairs or orchidopexies. Children with incarcerated hernias were excluded from this study due to the inflammation that sometimes occurs with incarceration. 

During surgery, tissue samples of white adipose tissue were taken from the visceral compartment (omentum majus) and the subcutaneous compartment (abdominal region). After acquisition, tissue samples were immediately stored in two separate storage tubes. One part of every tissue sample was stored in duplicate in plastic tubes with formaldehyde (10% buffered) for 12 h to be fixed and later embedded in paraffin blocks for further immunohistochemical and histological analysis. 

### 2.2. Anthropometric Measurements

Measurements of weight, height, and thigh and waist circumferences were made at the admission to the hospital before surgery. Height and weight were measured using standard techniques, a measuring tape and a weighing scale, respectively. Waist and thigh circumferences were measured with a measuring tape; waist/hip ratio was calculated. The waist circumference was determined at the level of the navel, and the thigh circumference was determined at the central part of the thigh. A BMI age- and sex-specific z-score was calculated according to the World Health Organization’s z-score reference data [20]. The BMI z-scores were interpreted as overweight in cases of >+1 SD and obese in cases of >+2 SD. Based on these definitions, there were 28 children with normal weights, 9 were overweight, and 8 obese. Considering these data, children were divided into two groups: normal weight (*n* = 28), and overweight and obese children together (*n* = 17). 

### 2.3. Blood Samples and Biochemistry

Blood samples were collected just before surgery and before general anesthesia, but following a 12 h fasting period. Serum glucose, triglycerides, total cholesterol, high-density lipoprotein (HDL) cholesterol, and low-density lipoprotein (LDL) cholesterol levels were measured using commercial enzyme kits (Department of Clinical Laboratory Diagnostics, University Hospital Osijek, Croatia), and the HDL/total cholesterol ratio was calculated. 

### 2.4. Adipose Tissue Morphology

Adipose tissue samples from the subcutaneous and visceral compartments were fixed in formaldehyde (10% buffered) as described before, then they were dehydrated and embedded in paraffin blocks for further analysis. Sections of embedded adipose tissue from both compartments were cut 6 μm thick using a Leica RM550 rotatory microtome (Leica, Vienna, Austria) and then stained with hematoxylin and eosin. To measure the adipocytes’ sizes in the sections of subcutaneous and visceral fat tissue, digital images were captured using an Olympus DP70 digital camera (Olympus, Tokyo, Japan) attached to a Zeiss Axioskop 2 MOT microscope (Carl Zeiss Microscopy, NY, USA). The digital images were stored in an uncompressed file format (tiff) for further analysis. All the images were acquired under the same conditions at 200× magnification. The adipocytes’ sizes were measured using the free online image software program Fiji [21], a distribution of ImageJ, with an Adiposoft plug-in [22], and were manually reviewed. 

### 2.5. Quantification of Collagen Content

Tissue sections of the SAT and VAT were stained with Masson’s trichrome stain for the detection of collagen content in the ECM. An analysis of the collagen content was performed using the free online image software program Fiji, a distribution of ImageJ, with digital images acquired under the same conditions at 200× magnification and stored as an uncompressed (tiff) file format for further analysis. The total collagen content was quantified using a modified method described by Ying Chan et al. [23]. The collagen content was reported as a percentage of Masson’s trichrome staining divided by the total tissue area in the field of view.

### 2.6. Immunohistochemistry

Immunohistochemistry for CD163 cells was performed using the BenchMark ULTRA fully automated workflow system (Ventana Medical System Inc., Oro Valley, AZ, USA) on both adipose tissue compartments. Anti-CD163 rabbit monoclonal antibodies (ab182422, Abcam, Cambridge, UK) that react with mouse, rat, and human samples were used as primary antibodies in a dilution of 1:100. CD163^+^ cells are considered to be M2-subtype macrophages. The slides were carefully examined to identify positive cells. The number of CD163^+^ cells was counted in mm^2^ of tissue in six randomly chosen areas on slides under 400× magnification by three independent researchers, with a histomorphometrical grid representing a surface area of 0.17 mm^2^ [24], and the average number was then calculated. The number of CLS was counted and normalized per the number of adipocytes as described by Fischer et al. [25].

### 2.7. Statistical Analysis 

All obtained data were analyzed using the IBM SPSS Statistics software (version 21, IBM Corporation, NY, USA). The Shapiro–Wilk test was used for normality checking; in cases of normal distribution, data were presented as the mean ± standard deviation (mean ± SD), and otherwise as the median and interquartile range. The differences between groups were determined using independent samples’ *t* tests or the Mann–Whitney U test. The difference was considered significant at *p* < 0.05. The correlation of normally distributed data was assessed with Pearson’s correlation coefficient *r* or with Spearman’s correlation coefficient ρ to determine the association between age, BMI z-score, collagen content, adipocytes’ surface area, and the number of CD163^+^ cells and CLS.

## 3. Results

### 3.1. Anthropometric Data

As expected, the BMI z-scores and the thigh and waist circumferences were significantly higher in the overweight/obese group compared with normal-weight children, but there was no statistical difference between the groups in terms of waist/hip ratio (Table 1). The children in the overweight/obese group were significantly older than the children in the normal weight group (7.29 ± 4.23 vs. 4.47 ± 2.64 years, *p* = 0.009).

### 3.2. Biochemical Analysis

No significant difference was measured in the serum glucose levels, testosterone, total cholesterol, HDL cholesterol, LDL cholesterol, and HDL/total cholesterol ratio between the groups (Table 1). Triglyceride serum levels were higher in the overweight/obese group compared to the normal-weight children (1.11 ± 0.37 vs. 0.84 ± 0.35 mmol/L, *p* = 0.021).

### 3.3. Adipocyte Morphology

Overall, the adipocytes’ measured surface area was larger in the subcutaneous than in the visceral fat depot (Table 1). Comparing groups, we noted no significant difference in VAT (*p* = 0.238), but, in subcutaneous fat, the average adipocyte’s surface area was significantly larger in the overweight/obese group compared to the normal-weight children (1031.15 ± 327.95 vs. 576.21 ± 191.95 µm^2^, *p* < 0.001).

### 3.4. Collagen Content in Adipose Tissue of Children

**Figure 1:**
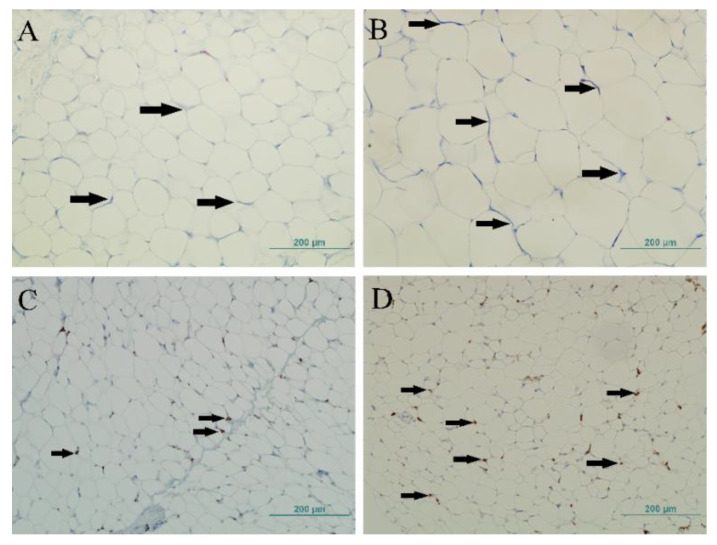
Masson’s trichrome staining of subcutaneous adipose tissue in (**A**) a normal-weight and (**B**) an overweight male child. The arrows indicate pericellular collagen (blue color). Magnification: 200×, scale bar 200 µm. The immunohistochemistry for CD163 cells in visceral adipose tissue in (**C**) a normal-weight and (**D**) an overweight male child. The arrows point to positively stained cells (macrophages). Magnification: 200×, scale bar 200 µm. The images were taken from representative samples.

Overweight/obese children had a significantly higher collagen staining in their SAT compared to normal-weight children (7.82 ± 4.21 vs. 5.46 ± 3.29%, *p* = 0.042; Figure 1A,B), but that was not observed in their visceral fat depot (Table 1).

**Figure 2:**
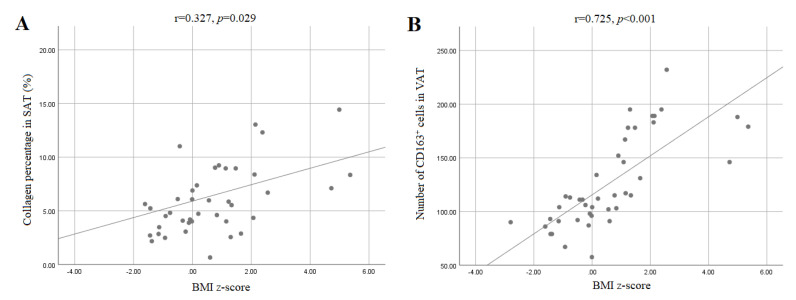
Pearson’s correlation between BMI z-score and (**A**) the collagen percentage in SAT and (**B**) the number of CD163^+^ cells in VAT. (**A**) The BMI z-score was positively associated with the collagen percentage in SAT (*r* = 0.327, *p* = 0.029). (**B**) The BMI z-score was also positively associated with the number of CD163^+^ cells in VAT (*r* = 0.725, *p* < 0.001). SAT—subcutaneous adipose tissue; VAT—visceral adipose tissue.

We observed that higher BMI z-scores were associated with increased collagen staining in SAT (*r* = 0.327, *p* = 0.029, Pearson’s correlation; Figure 2A), which persisted after a correction for age (*p* = 0.029). No such association was found in VAT. No correlation between age, adipocytes’ surface area and collagen content was found either in SAT or VAT, and there was no correlation after a correction for age. 

### 3.5. CD163^+^ Cells and CLS in the Adipose Tissue of Children 

More CD163^+^ macrophages were counted in the VAT than in the subcutaneous fat (Table 1), with significantly more CD163^+^ cells in the overweight/obese group’s visceral fat depot compared to the normal-weight group’s visceral fat depot (168.18 ± 33.13 vs. 98.57 ± 20.74, *p* < 0.001; Figure 1C,D). There was a positive correlation in the VAT between age (*r* = 0.553, *p* < 0.001), BMI z-score (r = 0.725, *p* < 0.001; Figure 2B), and adipocytes’ surface area (*r* = 0.325, *p* = 0.029; Pearson’s correlation) with older children and children with higher BMI z-scores and larger adipocytes having more CD163^+^ macrophages in their visceral fat depots. Whilst controlling for age, there persisted a positive correlation between the BMI z-score (r = 0.776, *p* < 0.001) and the number of CD163^+^ cells in the visceral adipose tissue. 

The number of CLS was higher in the group of overweight/obese children in both the SAT and VAT when compared to normal-weight male children (Table 1), which was statistically significant (*p* = 0.008 for SAT and *p* < 0.001 for VAT). The number of CLS correlated positively with BMI z-scores (SAT: *r_s_* = 0.335, *p* = 0.024; VAT: *r_s_* = 0.577, *p* < 0.001; Spearman’s correlation) and adipocytes’ surface area (SAT: *r_s_* = 0.344, *p* = 0.021; VAT: *r_s_* = 0.483, *p* = 0.001; Spearman’s correlation) in both adipose tissues, which persisted after a correction for age. 

## 4. Discussion

In this study, we examined white SAT and VAT collected from normal-weight and overweight children for adipocyte morphology changes and changes in collagen content as a marker of ECM remodeling. Male children were chosen because of the easier acquisition of research material due to their frequency of elective surgical procedures at a young age, and their later onset of puberty compared to females, so there would be no significant impact of sex hormones on adipose tissue development [26]. 

We found that the surface area of subcutaneous adipocytes was larger in the overweight/obese group of male children. The same group showed a higher collagen content in the ECM of SAT compared to the normal-weight male children, and there was a positive correlation between BMI z-scores and collagen staining, which persisted after a correction for age.

Studies in mice revealed that SAT adipogenesis occurs in early embryogenesis, and after birth, the number of adipocytes remains stable. On the other hand, visceral adipocytes mostly differentiate postnatally [27]. In humans, SAT forms from the 14th to the 24th gestational week from the head and neck to the caudal [28], and the number of adipocytes after the first year of age remains mostly stable until adolescence [29]. VAT remains small in extent until adolescence [30]. This could be the reason why we found changes only in the SAT, but not in the VAT, and the surface area of adipocytes increased, suggesting cell hypertrophy. Other authors also reported larger adipocytes in the SAT of obese children, suggesting that adipocytes’ hypertrophy and hyperplasia start at an early age [31]. Although the children in our overweight group were older, the testosterone levels did not differ significantly between groups, so we could exclude the effect of puberty.

Studies on adult humans and rodents showed that adipocytes’ hypertrophy promotes the leakage of free fatty acids, the elevation of proinflammatory cytokines, immune cell recruitment, hypoxia and fibrosis, and reduces insulin sensitivity [32]. In the overweight group of male children, we found significantly elevated triglyceride levels compared to normal-weight male children. A study by Arner et al. showed that individuals with adipocyte hypertrophy, which we have in this group, have a more adverse metabolic profile than those with hyperplasia [33]. Other authors state that LDL-cholesterol levels are predominantly influenced by genetic factors [34], other than triglycerides, which could be, in our case, influenced by environmental factors. Patients with cardiovascular disease and metabolic syndromes often present a lipid profile with elevated triglycerides, normal to mildly elevated LDL-cholesterol, and low HDL-cholesterol [35], and, in our study, we found elevated triglycerides and normal serum LDL-cholesterol, which could indicate the early start of a deteriorating metabolic state. The prevention and control of these risk factors could be beneficial at a pediatric age. 

In our overweight group, a higher collagen content in the ECM of SAT was found, with higher BMI z-scores associated with increased collagen staining. Tam et al. found in their study that overweight children had less collagen deposition in their adipose tissue [19]. Although our findings differ from these, in adults, studies have shown that collagen VI increases with BMI, and that obese adults with high collagen VI levels have an increased adipose tissue mass [36,37]. Some studies suggest that collagens are necessary for triglycerides’ accumulation in adipocyte progenitors in the early stages of differentiation into mature adipocytes [38,39]. It is also thought that greater collagen deposition restricts adipose tissue’s growth [40]. Our finding of larger SAT adipocytes in the overweight group could reflect an overall situation of dynamic tissue remodeling with changing contents of collagen in children, and maybe it can be hypothesized that the pathological nature of ECM fibrosis is more related to the type of collagen than to the extent of fibrosis, which we did not investigate. 

Collagen’s deposition in the ECM is a sign of fibrosis usually accompanied with an excessive production of ECM components by cells that are activated by inflammation [8]. There was no significant difference in the number of CD163^+^ cells in the SAT between groups, but CD163 is a marker for M2-polarized type macrophages, which are described as the anti-inflammatory type. In other studies, a higher number of CD68^+^ cells (proinflammatory macrophages) was visible in the SAT of obese adolescents [41], which we did not investigate in this study. The number of CD163^+^ cells in our study was higher in the VAT of the overweight group of male children and was positively correlated with age, BMI z-score, and adipocyte size. After a correction for age, our results showed a positive correlation between BMI z-scores and the number of CD163^+^ cells in the VAT, suggesting that age did not have much influence on this result. Our findings also support a higher presence of CLS in the overweight/obese children in both the SAT and VAT, which correlates with previous studies [31,42]. This could indicate that a low-grade inflammation is already present in childhood. The number of CLS was higher in the VAT than in the SAT, as reported by other authors [43], which could be because adipocytes in the VAT reach a critical size, triggering death, before their counterparts in the SAT, and we also found smaller adipocyte surface areas in the VAT. The positive correlation between adipocytes’ size and the number of CLS shows that macrophage infiltration is attracted by hypertrophic adipocytes [43,44]. 

Our study is limited with a relatively small sample size, but, given the number of available studies on children, it provides valuable insight into adipocytes’ morphology and collagen deposition in the adipose tissue of healthy male children, and poses questions that could be answered in the future. 

## 5. Conclusions

Changes in adipocytes’ morphology and increased collagen deposition can be observed in the SAT of young male children depending on their BMI z-scores, the prominent anti-inflammatory macrophage polarization in their VAT, and a higher number of CLS in both their SAT and VAT, which could present a future risk for metabolic-related diseases in obese individuals. 

## Figures and Tables

**Table 1:** Patients’ characteristics.

Group	Normal Weight	Overweight/Obese	*p*-Value
*n*	28	17	
Age (years)	4.47 ± 2.64	7.29 ± 4.23	0.009 *
BMI z-score	−0.37 ± 0.89	2.25 ± 1.40	<0.001 *
Thigh circumference (cm)	32.00 (28.00–36.88)	44.00 (35.00–56.00)	0.002 ^†^
Waist circumference (cm)	49.36 ± 11.96	61.29 ± 17.61	0.010 *
Waist/hip ratio	1.54 ± 0.41	1.40 ± 0.47	0.325 *
Glucose (mmol/L)	5.0 (4.40–5.80)	5.20 (4.60–6.10)	0.378 ^†^
Triglycerides (mmol/L)	0.84 ± 0.35	1.11 ± 0.37	0.021 *
Cholesterol (mmol/L)	4.22 ± 0.70	4.02 ± 0.86	0.418 *
HDL cholesterol (mmol/L)	1.45 ± 0.30	1.31 ± 0.24	0.087 *
LDL cholesterol (mmol/L)	2.56 ± 0.55	2.33 ± 0.71	0.236 *
HDL/total cholesterol ratio (%)	33.96 ± 5.56	35.18 ± 6.64	0.517 *
Testosterone (nmol/L)	0.23 (0.10–0.35)	0.34 (0.20–0.38)	0.171 ^†^
Adipocyte surface area (µm^2^)			
Subcutaneous AT	576.21 ± 191.95	1031.15 ± 327.95	<0.001 *
Visceral AT	354.52 ± 139.59	407.87 ± 153.69	0.238 *
Collagen percentage (%)			
Subcutaneous AT	5.46 ± 3.29	7.82 ± 4.21	0.042 *
Visceral AT	9.92 ± 3.78	10.76 ± 4.48	0.504 *
Number of CD163^+^ cells			
Subcutaneous AT	75.36 ± 34.26	84.29 ± 34.60	0.403 *
Visceral AT	98.57 ± 20.74	168.18 ± 33.13	<0.001 *
Number of CLS			
Subcutaneous AT	0.00 (0.00–1.00)	1.00 (0.00–2.50)	0.002 ^†^
Visceral AT	2.50 (2.00–4.00)	6.00 (4.00–7.00)	<0.001 ^†^

Data are presented as the mean ± standard deviation (mean ± SD) or the median and interquartile range. * Independent samples *t* test; ^†^ Mann–Whitney U test; BMI: body mass index; HDL: high-density lipoprotein; LDL: low-density lipoprotein; AT: adipose tissue; CLS: crown-like structures.
